# Perceived moral traits of others differentiate the neural activation that underlies inequity-aversion

**DOI:** 10.1038/srep43317

**Published:** 2017-02-23

**Authors:** Hironori Nakatani, Akitoshi Ogawa, Chisato Suzuki, Takeshi Asamizuya, Kenichi Ueno, Kang Cheng, Kazuo Okanoya

**Affiliations:** 1 Okanoya Emotional Information Project, Exploratory Research for Advanced Technology (ERATO), Japan Science and Technology Agency (JST), Kawaguchi, Saitama, 332-0012, Japan; 2 Cognition and Behavior Joint Research Laboratory, RIKEN Brain Science Institute, Wako, Saitama, 351-0198, Japan; 3 Center for Evolutionary Cognitive Science, Graduate School of Arts and Science, The University of Tokyo, Meguro-ku, Tokyo, 153-8902, Japan; 4 Department of Social Psychology, Graduate School of Humanities and Sociology, The University of Tokyo, Bunkyo-ku, Tokyo, 113-0033, Japan; 5 Department of Neurophysiology, Juntendo University, Bunkyo-ku, Tokyo, 113-8421, Japan; 6 Brain Science Institute, Tamagawa University, Machida, Tokyo, 194-8610, Japan; 7 Support Unit for Functional MRI, RIKEN Brain Science Institute, Wako, Saitama, 351-0198, Japan; 8 Laboratory for Cognitive Brain Mapping, RIKEN Brain Science Institute, Wako, Saitama, 351-0198, Japan; 9 Department of Life Science, Graduate School of Arts and Science, The University of Tokyo, Meguro-ku, Tokyo, 153-0198, Japan

## Abstract

We have a social preference to reduce inequity in the outcomes between oneself and others. Such a preference varies according to others. We performed functional magnetic resonance imaging during an economic game to investigate how the perceived moral traits of others modulate the neural activities that underlie inequity-aversion. The participants unilaterally allocated money to three partners (good, neutral, and bad). During presentation of the good and neutral partners, the anterior region of the rostral medial frontal cortex (arMFC) showed increased functional connectivity with the caudate head and the anterior insula, respectively. Following this, participants allocated more money to the good partner, and less to the bad partner, compared with the neutral partner. The caudate head and anterior insula showed greater activation during fair allocation to the good and unfair allocation to the neutral partners, respectively. However, these regions were silent during allocations to the bad partner. Therefore, the arMFC-caudate/insula circuit encompasses distinct neural processes that underlie inequity-aversion in monetary allocations that the different moral traits of others can modulate.

Value-based decision-making in a social interaction is based not only on self-interest but also on outcomes for other people. For example, considerations of fairness motivate us to reduce inequity in outcomes between oneself and others, and such social preference is known as *inequity-aversion*^1^,^2^,^3^, which is irrational for maximizing purely personal outcomes, but it would be rational in altruistic or cooperative behaviours towards others.

Inequity-aversion is associated with the concept of social value orientation^4^. *Prosocial value orientation* refers to a preference for maximizing equity in outcomes between oneself and others. Conversely, *individualistic value orientation* refers to a preference for maximizing outcomes only for oneself, even when one has a partner. Each individual has both types of social value orientations, and individual social preference of inequity-aversion is characterized by weighing both orientations.

Social preference, however, varies according to social factors. One salient factor is the perceived moral traits of the others. People tend to evaluate benefit for others positively or negatively based on whether they are fair or unfair, respectively^5^. For example, individuals take more risks to invest money with a fair partner than with an unfair partner in a trust game^6^,^7^. Individuals are more cooperative when they perceive their partners as being cooperative in a one-shot prisoner’s dilemma^8^.

Here, we investigate the neural functions that modulate individual preferences of inequity-aversion based on the moral traits of others. At least two types of functions might be involved in this modulation. One function is the *perception of the moral traits of others*. This function requires an evaluation of the person-specific psychological properties of others, such as personality traits and dispositions, based on their behaviour and intention in a social context. Neuroimaging studies suggest that the evaluation of the personality traits or intentions of others take place within the anterior region of the rostral medial frontal cortex (arMFC)^9^,^10^,^11^,^12^,^13^. The other function is the *valuation of inequity in outcomes between oneself and others* in a given social scenario. The inequity-aversion behaviour requires an evaluation of at least two different types of information, namely, one’s own outcome and the outcome differences between oneself and others; these are integrated into utility for decision-making. Studies about inequity-aversion show that the anterior insula and amygdala respond to unfair resource allocation between oneself and others^14^,^15^,^16^,^17^, and the caudate head represents the marginal utility of choice for money allocation^18^. The existence of several brain regions pertaining to perception of moral traits of others and valuation of inequity might suggest that these regions form a functional network when the perception of the moral traits of others modulates the inequity-aversion property. Therefore, we hypothesized that social modulation of inequity-aversion is established through interactions in the neural activities between regions for moral-perception and inequity-valuation^19^,^20^,^21^,^22^.

In the present functional magnetic resonance imaging (fMRI) study, the participants were scanned while they played a dictator game^23^. Participants unilaterally allocated money to oneself and a partner. There were three partners and each had good, neutral, or bad moral behaviours toward the participant. Before the dictator game, the participants engaged in a moral trait conditioning experiment. Therefore, the participants had impressions about the morals of the three partners. We evaluated cognitive factors that modulated the individual preferences of inequity-aversion with our model of decision utility that had two factors to represent inequity-aversion. The first factor was a gain to discount utility value due to inequity in money-allocation and the second factor was a threshold for perceiving inequity. To investigate the neural mechanisms that modulated inequity-aversion based on the moral traits of others, we analysed the neural activities in the moral-perception and the inequity-valuation regions, where the brain activity tracked decision utility in a trial-by-trial manner. Finally, we examined whether the functional connectivities of the neural activities between these two regions emerged while perceiving the moral traits of others or during the valuation of choice option for monetary allocation. In the former case, the perception of the moral trait of others modulated the activation of the inequity-valuation region in advance for subsequent decision-making. In the latter case, information about the moral traits of others was used to modulate the activation when the participants evaluated their utility, including the inequity between oneself and others. We show that the threshold for perceiving inequity is the dominant cognitive factor that modulates inequity-aversion and that activation in the moral-perception region interacts with activation in the inequity-valuation region in advance for the subsequent decision of monetary allocation between oneself and others.

## Results

### Experimental design

The study consisted of two experiments. The first one was the moral trait conditioning experiment outside of the MR scanner. Eighteen participants engaged in a stopwatch game with five observers whose photographs were presented on the screen. When the participant failed to stop the stopwatch at a target time, one observer encouraged (good person) and one observer disparaged the participant (bad person), and the other three observers commented neutrally (neutral person). The participants were conditioned toward the moral traits of the observers during this experiment.

**Figure 1:**
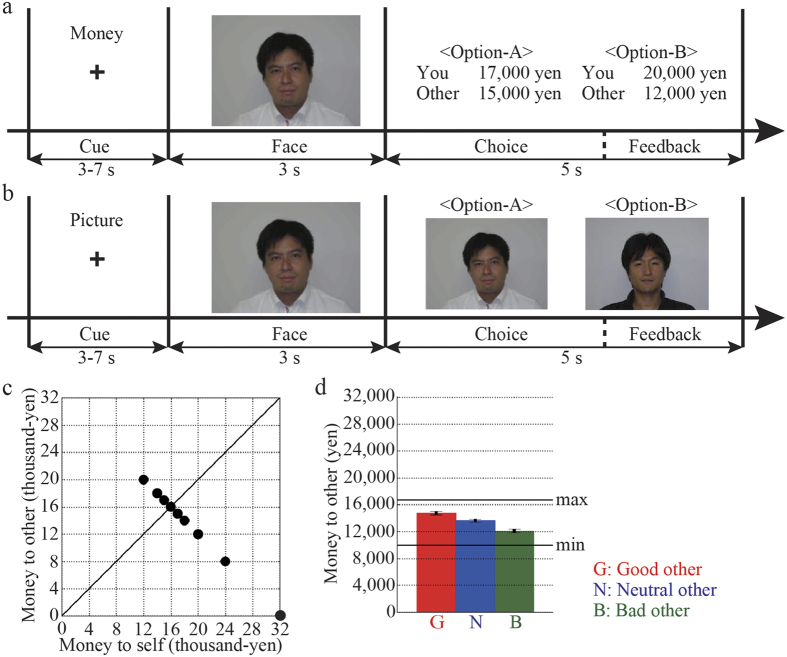
Experimental design and behaviour results. (**a**) The participants engaged in a dictator game involving a money-allocation task inside a magnetic resonance imaging (MRI) scanner. The participants played the role of the dictator. Three others played the role of receivers who each had a different moral trait, good, neutral, or bad. In each trial, the face of one of the three others was presented for 3 seconds during the face phase, after a 3–7 seconds task-cue phase. In the choice phase, a pair of choices was randomly presented. The participants allocated money to themselves and others by making a binary choice within 5 seconds. (**b**) In the face-choice task as the control task, two photographs were presented in the choice phase. The participants chose the photograph that was presented in the face phase within 5 seconds. (**c**) Each dot indicates an option for money allocation that was used in the choice phase of the game. The horizontal axis indicates the money obtained by the participants, and the vertical axis indicates the money that was allocated to others. Nine options of money allocation were used. (**d**) The group averages and standard errors of the money allocated to others by the participants. The money allocated to others was evaluated using a trial average of the money allocated to others. When the participants chose only options that allocated more money to others, the money to others was “max”. Conversely, when the participants chose only options that allocated less money to others, the money to others was “min”. G, N, and B indicate good, neutral, and bad others, respectively.

The second experiment was a money-allocation experiment that occurred inside of the MR scanner. We used a dictator game^23^ that is a unilateral money allocation task (this experiment is described in Methods section in the Supplementary materials). The good person, the bad person, and one of three neutral persons who appeared in the conditioning experiment were selected as partners for the second experiment in the MR scanner. The participants always allocated money to themselves and one of the partners in each trial. Each trial of the money-allocation task consisted of four phases, namely, a cue, face, choice, and feedback phases (Fig. 1a). A 3–7 second cue phase indicated the type of the task, either money-allocation (Fig. 1a) or a face-choice (Fig. 1b; see below for detail). One of three faces was presented as a partner for 3 seconds in the face phase. Two out of nine options for money allocation (Fig. 1c) were presented and the participants made a choice by pressing a corresponding (left or right) button within 5 seconds (choice phase), and then the chosen option was coloured green for the remaining period (feedback phase). We used all possible combinations of the nine options (i.e. 36 patterns) for each the personality traits of the others.

We introduced a face-choice task as a control task that did not require an evaluation of inequity in the outcomes between oneself and others. Each trial of the face-choice task consisted of four phases (Fig. 1b), similar to those in the money-allocation task. The same faces used in the money-allocation task were also presented in this task. In the face phase, a face was presented as a target. In the choice phase, target and non-target faces were presented instead of money-allocation options, and the participant was asked to choose the target face.

The money-allocation and face-choice tasks were performed in a random order. The faces were changed in every other trial. That is, after the same face was presented in a succeeding trial (i.e. a no-switch trial), a different face was presented in the subsequent trial (i.e. a switch trial).

### Effects of moral trait conditioning

**Figure 2:**
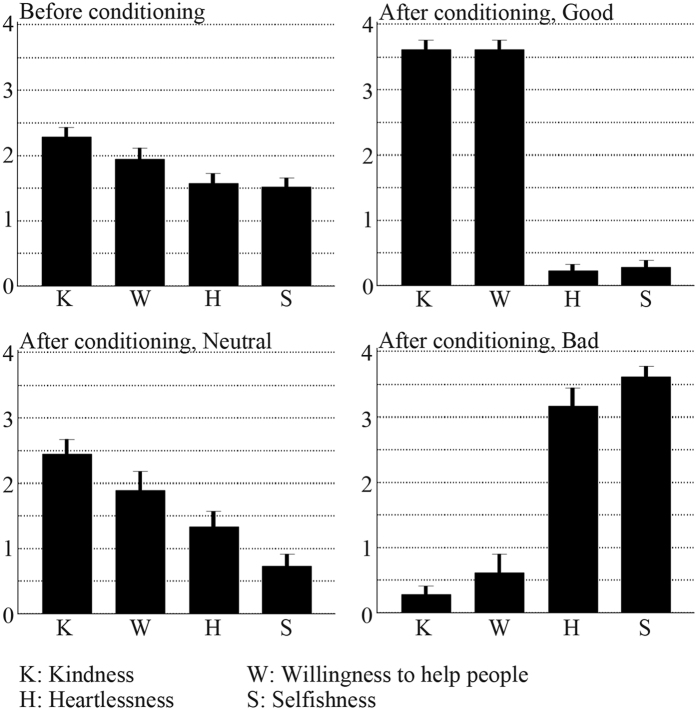
Conditioning effects of the moral traits of others. The impressions of others were evaluated by rating four questions using a five-point scale from zero (not at all) to four (very much). The questions were kindness (K), willingness to help people (W), heartlessness (H), and selfishness (S). The group averages and standard errors of ratings for each moral trait condition are presented.

Before and after the moral trait conditioning, the participants gave their impressions regarding a person’s traits using a printed questionnaire sheet on the following aspects: 1) thoughtfulness; 2) willingness to help people; 3) heartlessness; and 4) selfishness. They rated each personality on a five-point scale (zero - not at all; four - very much). We evaluated the effects of the conditioning on the participants by comparing the impression ratings before and after the moral trait conditioning with a two-way analysis of variance (ANOVA) (Fig. 2). The impression ratings of others were altered by the stopwatch game for the good partner (F_3,51_ = 79.57, *P* < *0.01*, interaction effect between moral trait conditioning and the questionnaire items) and the bad partner (F_3,51_ = 55.62, *P* < *0.01*, interaction effect). After the conditioning experiment, the participants perceived that the good and bad partner had positive and negative personalities, respectively. However, there was no effect on the impression rating for the neutral partner (F_3,51_ = 1.43, *P* > *0.05*, interaction effect; F_1,17_ = 3.21, *P* > *0.05*, main effect of conditioning). These results indicated that the participants had been successfully conditioned regarding the moral traits of the individuals who would be their partners in the money-allocation task that was conducted inside of the MR scanner.

### Effects of moral traits on money allocation

We evaluated the effects of moral traits on monetary allocation using a one-way ANOVA among the participants (F_2,17_ = 33.88, *P* < *0.01*, effect size *n* = *0.47*). A post-hoc paired *t*-test showed that the participants allocated more money to the good partner and less to the bad partner compared to the neutral partner (good versus neutral, t_17_ = 4.50; bad versus neutral, t_17_ = −4.87; *P* < *0.01*, with the Bonferroni correction; Fig. 1d). There was no difference between the reaction times for the moral trait conditions (good, 2.24 ± 0.07 s; neutral, 2.23 ± 0.08 s; bad, 2.15 ± 0.07 s; F_2,17_ = 2.45, *P* < *0.05, η* = *0.02*, one-way ANOVA among the participants). Thus, the cognitive load for monetary allocation was similar among the three moral trait conditions. No differences were observed in the monetary allocation properties between male and female participants (F_1,16_ = 1.36, *P* > *0.05*; two-way ANOVA, main effect of the gender), therefore we combined the data acquired from both sexes in subsequent analyses.

### Property of inequity-aversion

We compared our decision utility model with three others that characterized inequity-aversion behaviour. Model 1 is a pervasive one that was originally proposed by Fehr and Schmidt^1^, and assumes that a participant dislikes both advantageous and disadvantageous inequitable outcomes. The gains of difference in the outcome between oneself and others characterize the inequity-aversion property. However, Fehr and Schmidt’s model mainly focus on the ultimatum game and public goods game, and is not suitable for characterizing inequity-aversion behaviour in the dictator game; thus, we proposed a model (model 2). Our model states that not only the gain of difference but also the threshold of perceiving unfairness for advantageous inequity could characterize the inequity-aversion property. We expected that our model could characterize the inequity-aversion behaviours that outperform the original Fehr and Schmidt’s model, only if the participants ignored the small inequity in money allocation. In addition, we prepared two other models as controls. Model 3 was a composite between the Fehr’s model and ours that includes the gain and the threshold for advantageous and disadvantageous inequitable outcomes. Model 4 assumes that the participants are purely selfish and therefore do not care about any inequity. Details of these models are described in the Methods section. Based on the two alternative choice behaviours for monetary allocation, we evaluated the goodness of fit for the four models with Akaike’s information criterion (AIC).

**Figure 3:**
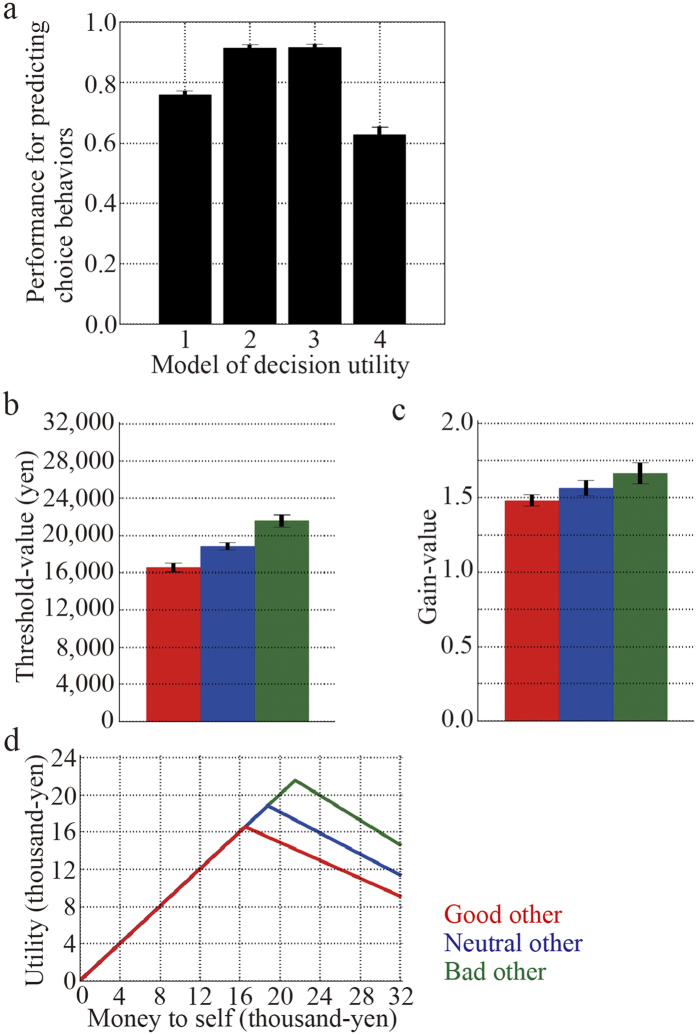
Decision utility properties. (**a**) Performances of four decision utilities for predicting behaviour choice. The numbers one, two, three, and four in the horizontal axis indicate models one, two, three, and four, respectively. These models are defined in the Methods section. (**b**) Group averages of the threshold value *θ* of the model 2 decision utility as defined by eq. (2). (**c**) Group averages of the gain value *α* of the model 2 decision utility. (**d**) Shape of the model 2 decision utility. The graph was plotted using the group averages of the threshold and gain values for each moral trait condition. Red, blue, and green indicate good, neutral, and bad others, respectively.

The results from model 2 and model 3 showed higher performances for predicting choice behaviours than those from models one and four (Fig. 3a). The AIC indicated that model 2 fit the behavioural data better than model 3 (t_17_ = −3.70, *P* < *0.01*; paired *t*-test; Table 1, Table S1); therefore, we used model 2 to analyse the fMRI data. The threshold for perception of unfairness was modulated by the moral traits of others that exhibited significant differences among moral trait conditions (F_2,17_ = 33.80, *P* < *0.01, η* = *0.47*; one-way ANOVA within the participants; Fig. 3b, Table S2). A post-hoc paired *t*-test indicated that the value was lower in the good partner condition and higher in the bad partner condition, as compared with the neutral partner condition (t_17_ = −4.69, *P* < *0.01* for good versus neutral; t_17_ = 4.81, *P* < *0.01* for bad versus neutral; paired *t*-test; with the Bonferroni correction, Table 2). In contrast, there was no significant difference in the value of gain among the moral trait conditions (F_2,17_ = 3.20, *P* > *0.05, η* = *0.09*; one-way ANOVA within the participants; Fig. 3c, Table S2). Therefore, the moral traits of others mainly modulated the threshold that influenced the inequity-aversion behaviour. The shapes of the decision utility functions for the three moral traits are shown in Fig. 3d.

### Brain regions engaged in moral-perception and inequity-valuation

**Figure 4:**
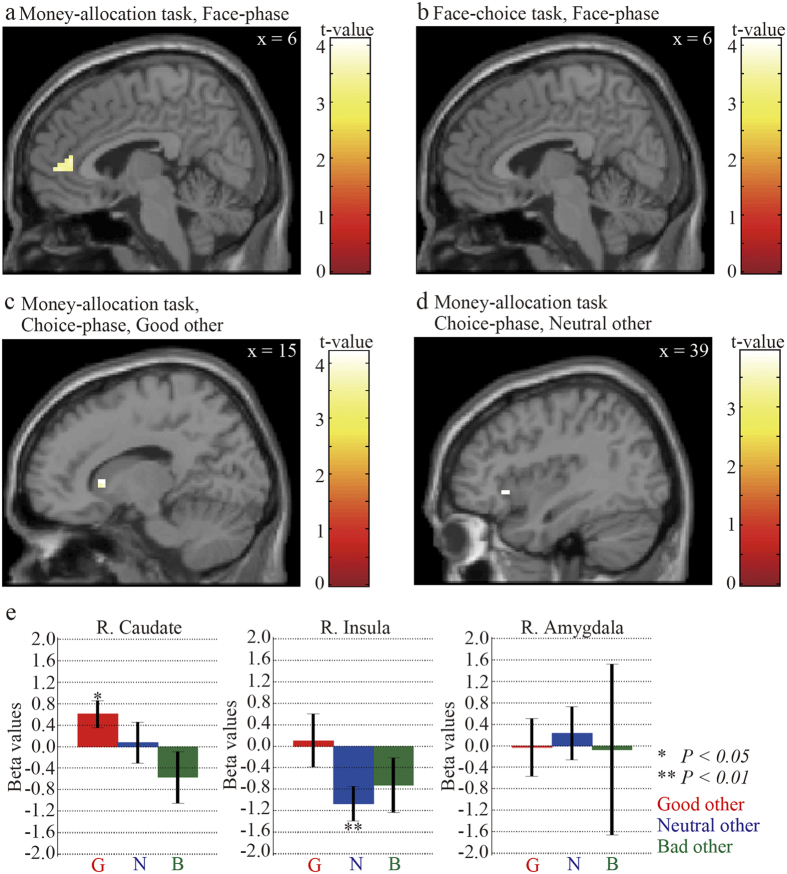
Moral-perception and inequity-valuation regions. (**a**) Activation of the anterior region of the rostral medial frontal cortex (arMFC) was associated with the perception of the moral traits of others in the money-allocation task. A sagittal view of activation within the right arMFC is illustrated. These examples are based on the t-statistic map of the contrast between switch and no-switch trials in the face-phase, with a significance threshold of P < 0.05 (SVC with the arMFC as the anatomical ROI). (**b**) Activation of the arMFC in the face-choice task. The contrast for the t-statistic map is the same as that in (**a**). (**c**) Activation of the caudate head was associated with the valuation of inequity-aversion in the good other condition of the money-allocation task. A sagittal view of activation within the right caudate head that was positively modulated by the decision utility function is shown (P < 0.05, small volume correction [SVC] with the caudate head as the anatomical region of interest [ROI]). (**d**) Activation of the anterior insula was associated with the valuation of inequity-aversion in the neutral other condition of the money-allocation task. A sagittal view of activation within the right anterior insula that were negatively modulated by the decision utility function is shown (P < 0.05, SVC with the caudate head as the anatomical ROI). (**e**) Beta values indicate strengths of the trial-by-trial modulations of the activation levels of the caudate head, insula, and amygdala by the decision utility value of the chosen option. Red, blue, and green bars indicate good, neutral, and bad moral traits of others, respectively. ** and * indicate 1% and 5% significance levels, respectively.

We first identified the region associated with the perception of the moral traits of others. A moral-perception region should be a part of the brain regions that are responsible for the evaluation of the personality traits of others. As the facial photographs were conditioned with the moral traits in our experiment, activation in such a region was expected to show adaptation on repeated presentation of an identical photograph^24^. We presented the same photograph on two successive trials, and compared the activation level of switch trials with that of no-switch trials at a single voxel level. We found that the right arMFC, which is known to be responsible for the evaluation of personality traits^9^,^10^,^11^,^12^,^13^, was involved in the process (*P* < *0.05*, positive effect of the switch of facial photographs presented in the two-way ANOVA within the participants). One factor of the two-way ANOVA was the switch of the facial photographs that were presented and the other factor was the moral trait of the partner. The family wise error (FWE) rate was corrected at the voxel level and a voxel threshold associated with a *P* < *0.05* for small volume correction (SVC)^25^ was t_102_ = 3.21 (positive effect of the switch of the facial photographs in the two-way ANOVA). The number of voxels in the anatomical region of interest (ROI) for SVC was 565, and the size of the voxel was 3 × 3 × 3 mm^3^ (see the “Definition of anatomical ROIs” section in the Supplementary materials; Table 2, Fig. 4a). In the face-choice task, the same contrast did not reveal any activity change in the right arMFC (Fig. 4b); this finding was probably due to the fact that the perception of the moral traits of others was not required in this task.

Next, we identified the region that was associated with the valuation of inequity in monetary allocation between oneself and others. The decision utility reflects the valuation of inequity in monetary allocation. If a region is responsible for the valuation of inequity, a trial-by-trial activation level in this region should be modulated by the trial-by-trial decision utility. Candidate brain regions for this function include the caudate head that represents the marginal utility for money allocation, the utility difference between the allocations that were chosen and not chosen^18^, and the anterior insula and the amygdala that respond to unfair resource allocations^14^,^15^,^16^,^17^. Our analyses revealed that the right caudate head was positively modulated by the value of the decision utility in the good partner condition (*P* < *0.05*, one-sample *t*-test; the FWE rate was corrected at the voxel level and a voxel threshold associated with a *P* < *0.05* for SVC was t_17_ = 3.53; the number of voxels in the anatomical ROI for SVC was 256; Table 3, Fig. 4c,e). The right anterior insula was negatively modulated in the neutral partner condition (*P* < *0.05*, one-sample *t*-test; FWE rate was corrected at the voxel level and a voxel threshold associated with a *P* < *0.05* for SVC was t_17_ = 3.68; the number of voxels in the anatomical ROI for SVC was 306; Table 3, Fig. 4d,e). The amygdala, however, did not display any significant modulation in any of the personality trait conditions (Fig. 4d).

### Functional connectivity between moral-perception and inequity-valuation regions

**Figure 5:**
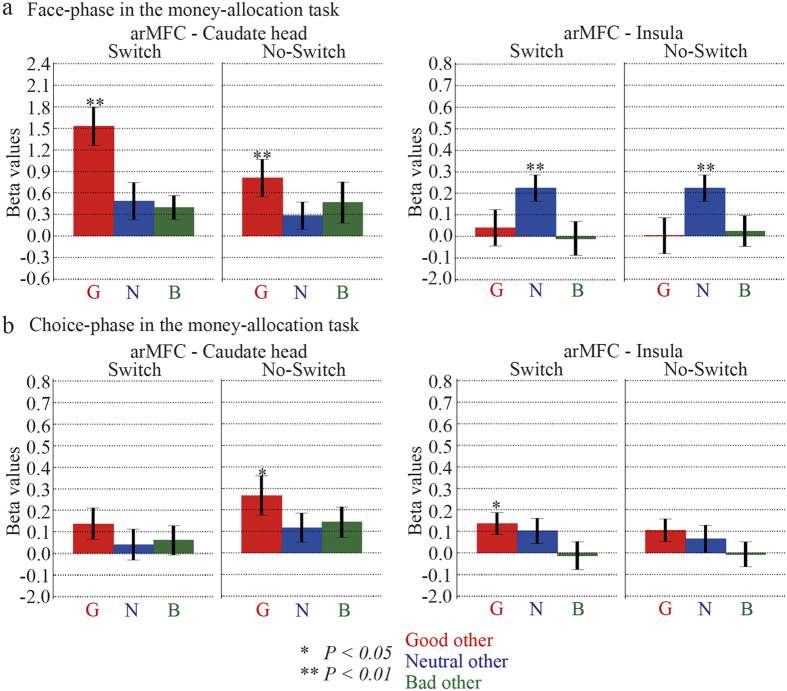
The strength of functional connectivity between the right arMFC and the right caudate head, and the right arMFC and the right insula in the money-allocation task. Beta values are shown that represent the strength of the connectivity between the right arMFC and the right caudate head and between the right arMFC and the right insula in the money-allocation task. (**a**) The face-phase. (**b**) The choice-phase. ** and * indicate 1% and 5% significance levels, respectively.

Distinct activation properties in the inequity-valuation region for the different moral traits of others might stem from an interaction between the moral-perception and inequity-valuation regions. We evaluated the functional connectivity between these regions using a generalized form of context-dependent psychophysiological interaction (gPPI)^26^. The seed region to calculate the strength of functional connectivity (i.e. beta values of the gPPI) was a 3 mm radius sphere ROI whose centre was a peak location of the arMFC (Table 2). The target regions to evaluate the strength of functional connectivity were two 3 mm radius sphere ROIs whose centres were peak locations of the caudate and insula (Table 3). Here, we defined the strength of functional connectivity as the mean beta values within a target ROI. The functional connectivity between the right arMFC and right caudate head was positive and significant only in the good partner condition (t_17_ = 5.63, *P* < *0.01*, switch trials of good other; t_17_ = 3.05, *P* < *0.01*, no-switch trials of good other; one-sample *t*-test; Fig. 5a). Moreover, functional connectivity between the right arMFC and right anterior insula was also positive and significant in the neutral partner condition (t_17_ = 3.54 *P* < *0.01*, switch trials of good other; t_17_ = 3.50, *P* < *0.01*, no-switch trials of good other; one-sample *t*-test; Fig. 5a).

In contrast, during the choice phase in the money-allocation task, the functional connectivity between the right arMFC and right caudate head in the good partner condition was significant only in the no-switch trials (t_17_ = 2.86, *P* < *0.05*, one-sample *t*-test; Fig. 5b). Moreover, the functional connectivity between the right arMFC and right anterior insula in the neutral partner condition was not significant in either the switch or no-switch trials. Further, in the face-choice task, significant connectivity was detected only in the switch trials during the face phase in the good partner condition (t_17_ = 3.72, *P* < *0.01*, one-sample *t*-test; Fig. S1). Therefore, the functional connectivity between the moral-perception and inequity-valuation regions appears to have been mainly established when the perception of the moral traits of others modulated the subsequent value-based decision-making.

## Discussion

We investigated how the moral traits of others modulated the behaviours and neural activities that underlie inequity-aversion. Behaviourally, the participants allocated more money to good partners and less money to bad partners compared to neutral partners. The moral traits of others mainly modulated the threshold of perceiving unfairness in the outcome difference between oneself and others. The fMRI results showed that the perceived moral traits of others based on a presentation of the partner’s face modulated arMFC-caudate/insula connectivity. Subsequently, the caudate head and anterior insula were differentially activated by the moral traits of others in the choice of monetary allocations. Altogether, our results suggest that while perceiving the moral traits of others, the arMFC may modulate the inequity-valuation system in advance of subsequent value-based decision-making.

In the decision utility model proposed by Fehr and Schmidt^1^ (Model 1), inequity is defined based on the difference in monetary allocations between oneself and others. Their model simply assumes that inequity aversion is linearly related to this difference, and the gain of the difference characterizes the inequity-aversion property. Although this simple linear assumption is appropriate for the ultimatum game and the public good game, it is not suitable for the dictator game^1^. In contrast, our decision utility model (model 2), which consisted of not only the gain of difference but also the threshold to perceive unfairness in the outcome difference, fit the behaviours of the participants better because the participants ignored small inequities in money allocation. Model 3 resulted in a similar performance as our decision utility function regarding the prediction of choice behaviours. The AIC analysis, however, revealed that our decision utility fit the participant behaviours better. Notably, the moral traits of others mainly modulated the threshold value in our decision utility function.

Inequity-aversion might be motivated by both the preference for fairness and the avoidance of unfairness. Fairness (i.e. decreasing inequity) has an intrinsically rewarding nature^27^, and unfairness (i.e. increasing inequity) has a negative impact on experienced rewards^1^,^28^. In the good partner condition, the peak value of the decision utility function corresponded to equal money allocation between oneself and others, and the value of the decision utility function positively correlated with the activation level in the caudate head. The caudate head is associated with the marginal utility of the participants’ choices, that is, the difference between the utility of allocations that were chosen and not chosen in a charitable donation task^18^. The caudate head is also associated with altruistic behaviour that results in great satisfaction^29^,^30^. In general, this region is also responsible for goal-directed behaviour^31^,^32^,^33^ through decision-making based on anticipated rewards^34^,^35^. Thus, fair money allocation to the good partner compared to the neutral or bad partner might have been rewarding for the participant. In other words, the participants likely willingly allocated money fairly to the good partner.

However, the decision utility for the neutral other negatively correlated with the activation level in the anterior insula. Neuroimaging studies have shown that the anterior insula responds to unfair money allocation in an economic game^14^,^18^, and to norm violation in social interactions^36^. In addition, this region is generally known to evaluate negative emotional states^37^,^38^. In our study, the lower value of the decision utility function in the neutral partner condition corresponded to unfair money allocation. When the two options were both unfair, the participants were forced to choose an unfair option for money allocation, and activation in the anterior insula was greater. Thus, unfair money allocation might have negatively affected the participants so that they avoided inequity in money allocation. In the bad other condition, activation was observed neither in the caudate, nor in the insula, and the participants allocated money unfairly to the bad other. The bad moral traits of others are likely to inhibit both types of motivation in order to avoid inequity.

We introduced photographs of faces that were conditioned with the moral traits used in the money-allocation task. The face is one of the most important features used to evaluate people in a social interaction^39^. The social category, identity, and emotions of individuals are evaluated by viewing their faces^40^,^41^. The arMFC is responsible for perceiving and mentalizing the social properties of others^9^. The arMFC, however, does not simply respond to the face of others but shows activation only when the mentalizing of others is necessary^12^. Consistent with this property, the arMFC showed larger activation irrespective of the types of the moral traits of others during the switch trials in the money-allocation task, where it was necessary to perceive the moral traits of others. The functional connectivity between arMFC and the inequity-valuation region was also detected in the face phase of the money-allocation task. Recognizing moral traits of others is necessary for proper behaviours in a social interaction, therefore the arMFC should play a role in modulating activities in a value-based decision-making system for subsequent social behaviour.

The present study has several limitations. First, we did not observe any brain activation that correlated with the threshold for the perception of inequity. Thus, we could not investigate how one’s own outcome and inequity in outcome allocation were represented and integrated into decision utility for inequity-aversion. Unlike decision utility, the threshold was effective only when one’s own outcome was greater than the threshold. The fact that the threshold was effective only in a small number of trials might have made it difficult to observe the associated brain activation.

A second limitation is that the brain region responsible for deciding monetary allocation was not fully identified. Although we showed that different brain regions represented decision utilities in different conditions of the morals of others, it was unclear which brain region integrated the decision utilities into a decision value regardless of the moral conditions of others. It is likely that decision utility is represented in the caudate head and/or in the anterior insula, but this idea is merely a speculation at present.

A third limitation is the possible contamination of blood-oxygen-level dependent (BOLD) responses between the face and choice phases because these phases in the experiment were not jittered. No jitter leads to collinearity in the BOLD responses between the face and choice phases. However, even if collinearity exists, it is possible to interpret the results^42^. We used a general linear model (GLM) to evaluate BOLD responses in individual data. Even if the collinearity between two parameters is high, the GLM provides us with unbiased estimates of parameters, but with inflated variances^42^; this means that averaged values of estimates across participants are close to true values of estimates. Our results were based on a group level analysis. Although inflated variances may reduce statistical power to detect any differences between parameters, the group averages of GLM-estimates of parameters reflected the group averages of BOLD responses. Therefore, we contend that our interpretations of the group level analysis data are valid.

The fourth limitation is the reliability of participants’ decision with regard to inequity-aversion. We did not provide participants with any real incentives for their money-allocation behaviours, thus the decision-making in the experiment was purely hypothetical. Such a hypothetical setting could have biased the results. In a study on value-based decision-making regarding purchasing consumer goods, valuation and brain activation were compared between hypothetical and real settings^43^. This study showed that participants overvalued goods, and brain activation levels were smaller in the hypothetical setting. However, this study also showed that the decision property in the hypothetical setting was comparable with that in the real setting when the bias was adjusted, and the same brain regions were associated with valuation in both setting. The authors concluded that the results from the hypothetical setting could be generalized to the real setting. Thus, we consider that our results regarding the neural activation that underlie inequity-aversion are reliable.

In conclusion, our results suggested that the moral traits of others modulate functional interactions between the arMFC and the caudate head, and between the arMFC and the anterior insula during the perception of others. These interactions impact distinct neural processes that underlie inequity-aversion during decision-making for monetary allocation.

## Methods

### Participants

We recruited 18 participants (six males and twelve females; average age [mean ± standard deviation], 22.7 ± 3.6 years), who were either recent university graduates or current students. The participants provided written informed consent before the experiment and were each paid 5,000 Japanese yen for their participation in the experiment. We did not provide participants with any real incentives that were associated with money-allocation behaviours. The Third Research Ethics Committee of RIKEN approved our procedures, and all methods were carried out in accordance with the Wako-3 21–14 institutional guideline.

### Moral trait conditioning experiment

The experiment consisted of pre and post conditioning impression ratings and playing a stopwatch game for moral trait conditioning.

In the pre-conditioning impression rating, the participants viewed photographs of the faces of five male individuals and gave impressions of them on a five-point scale questionnaire of traits such as thoughtfulness, willingness to help people, heartlessness, and selfishness.

The participants then engaged in a stopwatch game and were asked to stop it precisely at the target time displayed on a 20-inch flat-panel monitor (Cinema Display, Apple, Cupertino, CA, USA). In each trial, the target time was presented until the participants clicked the button to start the stopwatch. They were asked to click the button again to stop the stopwatch within ±0.3 seconds of the target time. Feedback on the result, either as a success or failure, was immediately provided. Then, five photographs of faces that were identical to those used in the preconditioning impression rating were presented. Moral traits were assigned to each photograph representing a fictional partner, and one good, three neutral, and one bad fictional partner were randomly assigned among the participants. When a participant failed to stop the stopwatch, the good and bad partner gave encouraging and disparaging text messages, respectively. One of the randomly selected neutral partners also gave an encouraging message. As each of the neutral partners gave the message only once after every three fails, the participants’ impressions of them were expected to be weaker than those of the good and bad partners. There were 40 trials. The average success rate was 0.57 ± 0.02 (mean ± standard error).

For the post conditioning impression rating, the participants rated impressions of the five partners using the same printed questionnaires as those used before the game.

### Decision utility functions

We developed a new decision utility function based on the Fehr and Schmidt model^1^. We eventually defined and compared four decision utility functions, including two additional controls, to characterize the inequity-aversion behaviour. Each model was a linear utility function of *xS* and *xO*, which was the money that the participant allocated to him or herself and to others, respectively.

Fehr and Schmidt’ model (model 1)^1^ assumes that the participant disliked an inequitable outcome. The gain of difference in the outcomes between oneself and others characterizes the property of inequity-aversion.


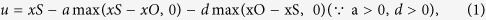


in which *u* was the decision utility of a chosen option for money allocation (Fig. 1c), the term *a*(*xS* − *xO*) reduced the decision utility by advantageous inequity, and *α* was the gain of discounting. The term *d*(*xO* − *xS* reduced the decision utility by disadvantageous inequity and *d* was the gain of discounting. Note that Fehr and Schmidt’s model mainly focuses on the ultimatum game and the public goods game, and it is not suitable for the dictator game^1^.

Our model (model 2) assumed that not only the gain of difference but also the threshold for perceiving advantageous inequity characterized the inequity-aversion property.


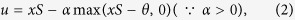


in which the term *α*(*xS* − *θ*) reduced the decision utility by perceived advantageous inequity, *α* was the gain, and *θ* was its threshold.

Model 3 was a compromise between the first and second models. It included the gain and threshold for advantageous and disadvantageous inequitable outcomes.


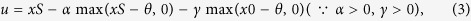


in which the term *γ*(*xO* − *θ*) discounted decision utility by perceived disadvantageous inequity and *γ* was the gain.

Model 4 assumed that the participants were purely selfish and did not care about an inequitable outcome.


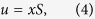


We estimated the model parameters separately for each moral trait based on the behaviour choices (see “Parameter estimation of the decision utility function” section in the Supplementary materials). We then selected the model that best fit to the behavioural data based on Akaike’s information criterion (AIC).

### Magnetic resonance imaging (MRI) scanning procedure

We conducted fMRI experiments on a 4T whole-body MRI system (Agilent Technologies, Santa Clara, CA, USA) using a transverse electromagnetic volume coil as the transmitter (Takashima Seisakusho, Tokyo, Japan) and a 16-array coil as the receiver (Nova Medical Inc., Wilmington, MA, USA). We acquired 36 continual slices of functional images per volume (FOV = 208 × 208 mm; 64 × 64 matrix; slice thickness = 4 mm) using a four-segment echo planar imaging (EPI) pulse sequence (echo time [TE] = 20 milliseconds; flip angle [FA] = 65.3 degrees). Slices were tilted 30 degrees from the anterior commissure-posterior commissure plane to the forehead. The TSENSE^44^,^45^ reconstruction (acceleration factor of 4) resulted in an effective volume repetition time (TR) of 1.17 seconds. We also recorded respiratory and cardiac signals using a pressure sensor and a pulse oximeter respectively, and removed these physiological fluctuations from the EPI images^46^.

### fMRI data analysis

We used SPM8 (Wellcome Trust Centre for Neuroimaging, Institute of Neurology, University College, London, UK) for data pre-processing and analysis of the functional images. In pre-processing, all EPI data were realigned to the mean image across all images and were resampled to 3 × 3 × 3 mm^3^. The slice-timing of the realigned EPI data were corrected with the middle slice of the volume as a reference timing. All slice-timing corrected EPI data were normalized to the EPI template in the Montreal Neurological Institute space, and then spatially smoothed using a Gaussian filter (full width at half maximum [FWHM] = 8 mm). A high-pass filter (128 s) was used to remove the low-frequency drift in the time course.

Based on our assumption regarding the perception of moral traits of others and valuation of inequity aversion, we focused our analysis on the arMFC, caudate head, anterior insula, and amygdala^9^,^10^,^11^,^12^,^13^,^14^,^15^,^16^,^17^,^18^, and defined anatomical ROIs for these regions (see Methods section in the Supplementary materials). We used a general linear model (GLM) to analyse individual data and performed a random effects analysis at the group level. To assess the statistical significance of the contrast, we restricted regions for analysis to an anatomical ROI with the WFU Pick Atlas software (ANSIR Laboratory, Wake Forest University School of Medicine, USA) and applied a small volume correction (SVC)^25^.

To analyse the region associated with the perception of the moral traits of others, we merged three moral trait conditions in the GLM. The GLM included five regressors, two were associated with the moral traits perception of others, i.e. the face phases in both switch and no-switch trials, and the remaining three were the cue, the choice, and the feedback phases. The GLM also included nuisance regressors to remove head motion artefacts. The nuisance regressors were the head motions extracted at the realignment, and had six parameters regarding head motions, i.e. x-, y- and z-directions and pitch-, roll- and raw-rotations. We contrasted switch with no-switch trials to identify regions related to moral trait perception because a brain region that is responsible for such a function would show a reduced response to repeated presentations of the same facial photograph because of fMR-adaptation^24^.

We applied a parametric modulator of task regressors to the GLM to identify inequity-valuation regions. The modulator represented the decision utility value of a chosen option for money allocation. As we were interested in how the moral traits of others modulated the neural activities related to inequity aversion, three moral trait conditions were separately included in the GLM. The GLM included five regressors; one was the parametric modulator of the choice phase, and the remaining four were the cue, face, choice, and feedback phases. The GLM also included head motion as nuisance regressors to remove the head motion artefacts.

When neural activities underlying inequity aversion were modulated by the moral traits of others, the region for moral-perception might interact with that for inequity-valuation. We evaluated the functional connectivity between these regions with a generalized form of context-dependent psychophysiological interaction (gPPI)^26^. The GLM for the gPPI separately included the three moral trait conditions. The model to calculate gPPI consisted of three factors, seed time series, task regressors, and products of task regressors and seed time series. The seed region of time series was a 3 mm radius sphere ROI whose centre was a peak location of the arMFC (Table 2). We used 15 task regressors. When we calculate gPPI in the face-phase, 12 regressors were established for each condition of the face phase (moral traits {good, neutral, bad} × switch of presented face {switch, no-switch} × task condition {money-allocation, face-choice}). The remaining three regressors were cue, choice, and feedback for all conditions. When we calculate gPPI in the choice phase, 12 regressors established were for each condition of the choice phase and the remaining three regressors were cue, face, and feedback for all conditions.

## Additional Information

**How to cite this article**: Nakatani, H. *et al*. Perceived moral traits of others differentiate the neural activation that underlies inequity-aversion. *Sci. Rep.* **7**, 43317; doi: 10.1038/srep43317 (2017).

**Publisher's note:** Springer Nature remains neutral with regard to jurisdictional claims in published maps and institutional affiliations.

## Supplementary Material

Supplementary Information

## Figures and Tables

**Table 1:** The second model fit money allocation behaviour better than the other models.

Model	Number of parameters	Average values of AIC (*μ* ± se)
Model 1	2	35.7 ± 1.3
Model 2	2	19.7 ± 1.6
Model 3	3	20.9 ± 1.5
Model 4	0	48.9 ± 0.8

The group mean and standard error (*μ*: mean, se: standard error) of the average Akaike’s information criterion (AIC) values are provided across each partner moral trait for each model.

**Table 2:** Summary of the results of brain activation associated with the perception of the moral traits of others.

Region	Side	MNI coordinates			z-value	Number of voxels
		x	y	z		
^1^arMFC	R	6	50	7	3.46	22

^1^Retained after small volume correction (SVC) (*P* < *0.05*) with the anterior region of the rostral medial frontal cortex (arMFC) as the anatomical region of interest (ROI). The number of voxels in the ROI was 565, and a voxel threshold associated with SVC (*P* < *0.05*) was t = 3.21.

**Table 3:** Summary of the results of brain activation associated with the choices for money allocation.

Region	Side	MNI coordinates			z-value	Number of voxels
		x	y	z		
^1^Caudate head	R	15	20	4	3.43	7
^2^Anterior insula	R	39	23	−5	3.28	2

^1^Retained after SVC (*P* < *0.05*) with the caudate head as the anatomical ROI. The number of voxels in the ROI was 256, and a voxel threshold associated with SVC (*P* < *0.05*) was t = 3.53.

^2^Retained after SVC (*P* < *0.05*) with the anterior insula as the anatomical ROI. The number of voxels in the ROI was 306, and a voxel threshold associated with SVC (*P* < *0.05*) was t = 3.68.
