# Supplementary material for: Perceived moral traits of others differentiate the neural activation that underlies inequity-aversion
Source: Sci Rep. 2017 Feb 23;7:43317. doi: 10.1038/srep43317 (PMC5322339; doi:10.1038/srep43317)
Supplement: Supplementary Information [file srep43317-s1.pdf]

Supplementary materials

Perceived moral traits of others differentiate neural activations that underlie inequity-aversion

Hironori Nakatani, Akitoshi Okanoya, Chisato Suzuki, Takeshi Asamizuya, Kenichi Ueno, Kang Cheng, and Kazuo Okanoya

Methods

Cover story for the money-allocation experiment. In the conventional dictator game, there are individual differences in the amounts of money allocated[1]; some are motivated to allocate money to others while others are not. Therefore, we made a cover story to motivate all participants to allocate money to others. The participant was asked to imagine him or herself as a coffee shop manager that planned to hire a part-time employee to run the shop on the upcoming 2-day weekend. The total salary budget for the manager and part-time employee was 32,000 Japanese yen (100 Japanese yen

approximately correspond to 1 USD). The participant was asked to determine the salaries for himself/herself and the employee.

Parameter estimation of the decision utility function. To estimate the decision utility function parameters, we defined a negative log-likelihood function and estimated the model parameters that minimized the log-likelihood with a brute-force search method on a grid segmentation of parameter space. The parameters to be estimated were α and d in eq. (1) in the "Decision utility functions" section for model one, α and θ in eq. (2) for model two, and α, γ and θ in eq. (3) for model three.

Definition of anatomical ROIs. We defined anatomical ROIs for the arMFC, caudate head, anterior insula, and amygdala for small volume correction analysis using the automated anatomical labeling (AAL) structural ROIs in MarsBar software[2].

To define an anatomical ROI within the arMFC, we first defined that of the MFC. The MFC consisted of the cingulum_ant-ROI, cingulum_mid-ROI, and frontal_sup_medial-ROI of the AAL. Based on Fig. 3 in a previous study by Amodio

and Frith[3], we limited the anatomical ROI of the MFC within the region of $y > 40$ and $0 < z < 40$ to define the anatomical ROI within the arMFC. The number of voxels in the right arMFC and the left arMFC were 565 and 616, respectively.

We defined an anatomical ROI within the caudate head by limiting the caudate-ROI of the AAL within $y > 0$ based on Robinson et al.[4]. The number of voxels in the right caudate head and the left caudate head were 256 and 245, respectively.

We defined an anatomical ROI within the anterior insula by limiting the insula-ROI of the AAL within $y > 3$, based on a study by Lancaster et al.[5]. The number of voxels in the right anterior insula and the left anterior insula were 306 and 332, respectively.

We used the amygdala-ROI of the AAL as an anatomical ROI of the amygdala. The number of voxels in the right amygdala and the left amygdala were 73 and 65, respectively.

References

1. Fehr, E., Schmidt, K. M. A theory of fairness, competition, and cooperation. *Q. J.*

Econ. 144, 817-868 (1999).

2. Brett, M., Anton, J. L., Valabregue, R., & Poline, J. B. Region of interest analysis using an SPM toolbox [abstract] Presented at the 8th International Conference on Functional Mapping of the Human Brain, June 2-6, 2002, Sendai, Japan. Available on CD-ROM in Neuroimage, Vol 16, No 2.
3. Amodio, D. M. & Frith, C. D. Meeting of minds: the medial frontal cortex and social cognition. *Nat. Rev. Neurosci.* 7, 268-277 (2006).
4. Robinson, J. L. et al., The functional connectivity of the human caudate: an application of meta-analytic connectivity modelling with behavioural filtering. *Neuroimage* 60, 117-129 (2012).
5. Lancaster, J. L. et al., Bias between MNI and Talairach coordinates analysed using the ICBM-152 brain template. *Hum. Brain Mapp*. 28, 1194-1205 (2007).

Table S1.

The second model fit money allocation behaviour better than the other models. The group mean and standard error (μ: mean, se: standard error) of AIC values are given of each model are provided for each type of moral trait partner (others).

Model	Others	Number of parameters	Negative log likelihood (μ ± se)	AIC (μ ± se)
	Good	2	11.4±1.0	26.8±2.0
Model 1	Neutral	2	20.9±0.9	45.8±1.8
	Bad	2	15.2±1.0	34.4±2.0
	Good	2	8.8±0.8	21.6±1.6
Model 2	Neutral	2	6.9±1.3	17.8±2.6
	Bad	2	7.9±0.9	19.8±1.8
	Good	3	8.1±0.9	22.2±1.8
Model 3	Neutral	3	6.5±1.2	19.0±2.4
	Bad	3	7.8±0.9	21.6±1.8

	Good	0	26.7±0.3	53.4±0.6
Model 4	Neutral	0	21.5±1.0	43.0±2.0
	Bad	0	25.1±0.4	50.2±0.8

Table S2.

The parameters of decision utility were modulated by the partner moral traits. Shown are the group mean and standard error (μ: mean, se: standard error) of the gain α of the difference in the outcomes between oneself and others, and threshold θ of the perception of the unfairness in the money-allocation that reduce the decision utility value in eq. (2).

	Gain α (μ ± se)	Threshold θ (μ ± se)
Good	1.48 ± 0.03	16640 ± 320
Neutral	1.56 ± 0.05	18880 ± 320
Bad	1.66 ± 0.07	21400 ± 640

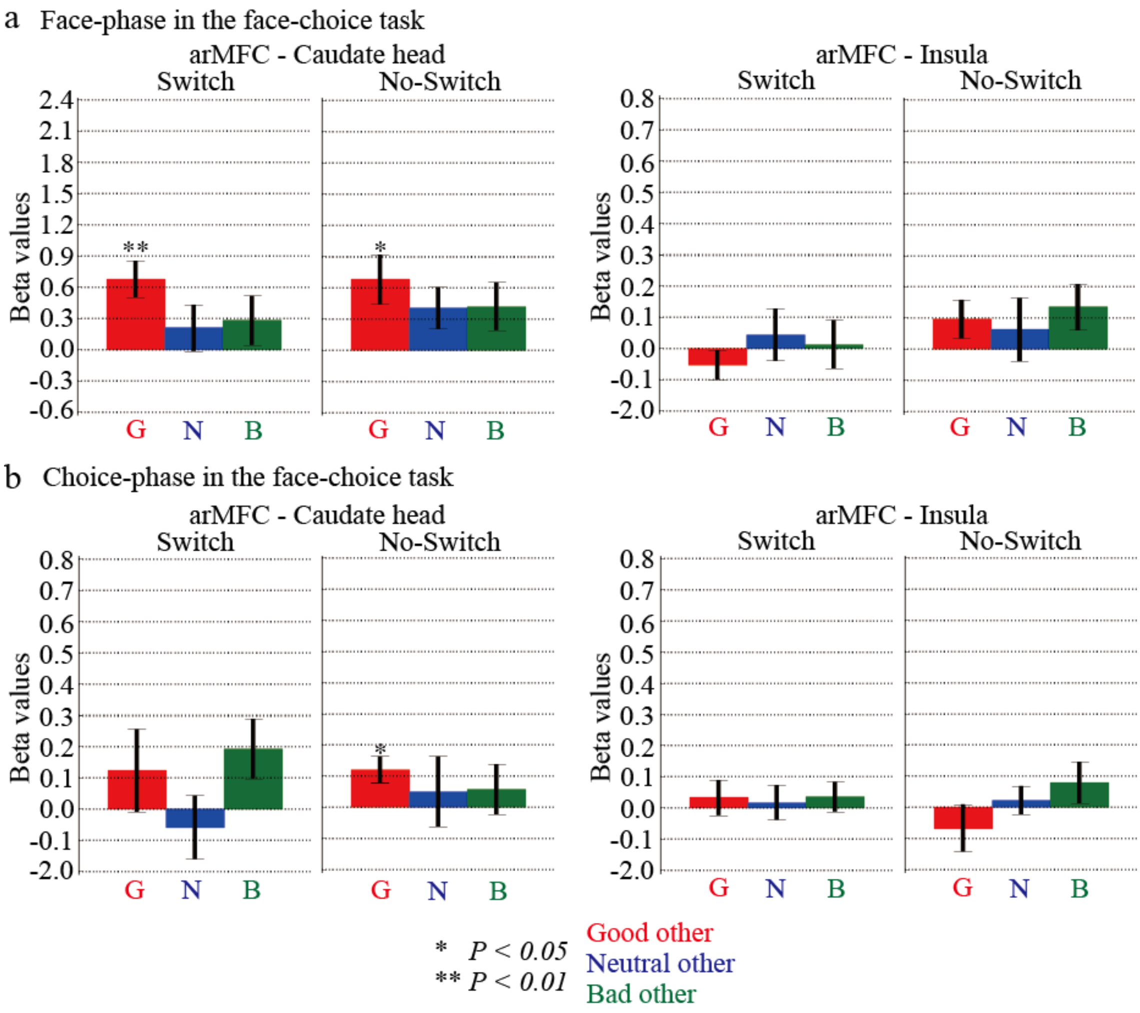

Fig. S1

Fig. S1. The strength of the functional connectivity between the right arMFC and the right caudate head, and between the right arMFC and the right insula in the face-choice task. Beta values that represent the strength of the connectivity between the right arMFC and the right caudate head and between the right arMFC and the right insula in the face-choice task are shown. Unlike the money-allocation task (Fig. 5), the right

arMFC and the right insula do not show significant strong functional connectivity in the face-phase of the face-choice task. a) The face-phase. b) The choice-phase. ** and * indicate 1% and 5% significance levels, respectively.
